# Sirtfoods: New Concept Foods, Functions, and Mechanisms

**DOI:** 10.3390/foods11192955

**Published:** 2022-09-21

**Authors:** Otobong Donald Akan, Dandan Qin, Tianyi Guo, Qinlu Lin, Feijun Luo

**Affiliations:** 1 Hunan Provincial Key Laboratory of Grain-Oil Deep Process and Quality Control, Hunan Provincial Key Laboratory of Forestry Edible Resources Safety and Processing, College of Food Science and Engineering, Central South University of Forestry and Technology, Changsha 410004, China; 2 Microbiology Department, Faculty of Biological Science, Akwa-Ibom State University, Ikot Akpaden, Uyo 1167, Nigeria

**Keywords:** Sirtfoods, SIRT, SIRT-modulating compound, polyphenol, longevity, eubiosis, gut microbiota

## Abstract

Sirtfood is a new concept food that compounds diets that can target sirtuins (SIRTs). SIRTs are nicotinamide adenine dinucleotide (NAD^+^)-dependent deacylases and ADP-ribosyltransferases (enzymes). SIRTs are mediators of calorie restriction (CR) and their activation can achieve some effects similar to CR. SIRTs play essential roles in ameliorating obesity and age-related metabolic diseases. Food ingredients such as resveratrol, piceatannol, anthocyanidin, and quinine are potential modulators of SIRTs. SIRT modulators are involved in autophagy, apoptosis, aging, inflammation, and energy homeostasis. Sirtfood proponents believe that natural Sirtfood recipes exert significant health effects.

## 1. Introduction

The search for wholesome food patterns that can ameliorate metabolic syndromes resulting from malnutrition and over-nutrition is gaining ground [1,2]. Studies on health-promoting food components show broad interest in exploring novel dietary patterns. The aim is to proffer accurate and individualized guidance for patients of different sub-health. Remarkably, polyphenols, dietary fibers, and other functional plant-sourced components have positive health effects in many cell and animal experiments, as well as clinical trials [3,4,5].

Dieticians have proposed different dietary patterns to combat the growing number of diseases caused by Western diets. However, their reliance on scientific data, as well as inappropriate translation and experimental results, may make recommended diets and intake regimes disadvantageous to consumers in the long run. A novel concept food, ”Sirtfood,“ has emerged and modulates SIRT (1–7) genes to affect health [6,7].

Mammalian SIRT enzymes are nicotinamide adenine dinucleotide (NAD^+^)-dependent deacylases and ADP-ribosyltransferases that regulate and mediate calorie restriction pathways [8,9,10,11]. In this paper, we present the health-promoting mechanisms of natural Sirtfood components. Sirtfoods and their metabolites have no side effects. They can modulate SIRTs, have strong antioxidant activity, and interact with gut microbes and metabolic components. Sirtfoods interfere with many signaling pathways and target genes and exert biological effects through synergistic networks. Sirtfoods are effective superfoods for the treatment and prevention of some diseases.

## 2. SIRT Health Functions

### 2.1. SIRT Genes

The first discovered silent information regulator 2 (Sirt2) gene prototype was the mating-type regulator 1 (MAR1); it was found in yeast and suppressed ribosomal DNA recombination, silenced genes, and regulated replicative lifespan [12]. Sirt2 proteins are universally present in all living organisms but become functionally complex in complex organisms [13]. The core of the SIRT structure consists of three parts: a large domain called the Rossman fold, characterized as the nicotinamide adenine dinucleotide (NAD^+^)-binding unit; a smaller Zn^2+^-binding motif domain; and an α-helical region that differentiates the seven mammalian SIRT family members [14]. SIRTs cleave acetyl groups from acetylated lysine in histones and other substrate proteins—resulting in condensed and inactive chromatin structures and gene silencing.

SIRT bioactivities are mainly controlled by dynamic changes in NAD^+^ levels and the NAD/NADH ratio, enabling cells to accept and donate electrons during essential reactions. NAD^+^ is deacetylated through a two-step reaction. In the first step, NAD^+^ is consumed to yield nicotinamide (NAM) and a 2- and 3-O-acetyl-ADP-ribose (OAADPr) mix. The second step releases deacetylated substrates [15]. The recognition of NAD^+^ has partly been due to the discovery of the mammalian SIRT family genes. SIRTs 1–7 genes’ differential subcellular localization, catalytic domain (CD) sequence, N/C-terminal do-main length, catalytic actions, and dependence on changes in NAD^+^ level allow for rapid regulation of many bioactivities in multiple tissues [9,12,16]; Different phytochemicals can modulate different SIRT members, and different SIRT members have different locations and substrates in cells, meanwhile, SIRT members have discrepancy functions and also have similar effects [7,17,18], see Table 1.

**Figure 1:**
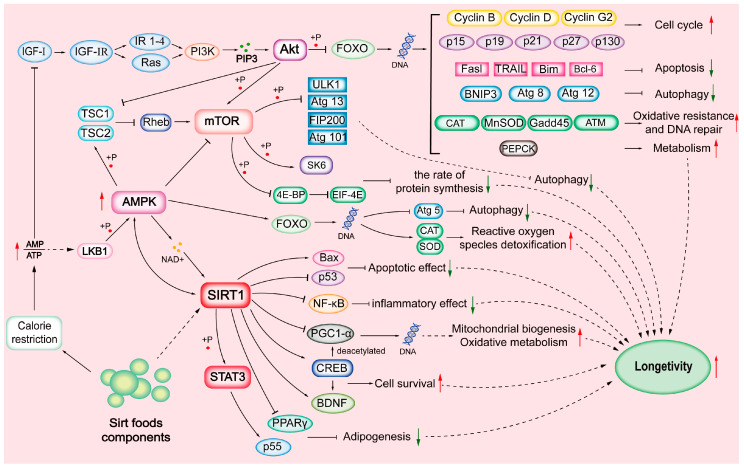
Sirtuin (SIRT)-mediated mechanisms.

SIRTs are substrate-specific and are activated during CR. SIRTs are involved in cell proliferation, differentiation, DNA damage repair, genome stability, life extension, energy homeostasis, stress resistance, organ development, aging, cancer, tissue regeneration, inflammation, neuronal signaling, and circadian rhythms [9,16,19]; see Table 1. CR condition decreases cellular energy status, increases the AMP:ATP ratio, and activates the AMP-activated protein kinase (AMPK) pathway. There is growing interest in targeting SIRTs through diets, which promote health primarily by activating the NAD^+^/SIRT pathway and their downstream components [17,20]; see Figure 1.

### 2.2. SIRTs and Oxidative Stress

Normal cellular functions generate reactive oxygen species (ROS) in the mitochondria. Excessive production and accumulation of ROS impair cellular viability through DNA structure damage and oxidations of protein, fatty acids, and lipids [15,16]. However, SIRT1 and SIRT3 activations are involved in mitohormesis [21]. Here, mitochondrial activities are regulated by deacetylating relevant proteins to stimulate mitohormetic responses—small harmful ROS activations that increase mitochondria number and actions to combat injury. In a recent study, the oral administration of piceatannol (10 mg/kg BW/day) attenuated hepatic and renal mitochondrial oxidative stresses in male albino rats. Piceatannol induces the production of antioxidative response elements (SOD, CAT, GSH-Px, and GR) and suppresses the expression levels of pro-inflammatory (TNF-α, IL 6) and apoptotic (cytochrome c, caspase-3) biomarkers. Piceatannol can exert antioxidation effects through SIRT1/p38/AMPK/PGC-1α pathway activation [22]. Genistein treatment suppresses the production levels of reactive oxygen species (ROS) and malondialdehyde (MDA) in human umbilical vein endothelial cells (HUVECs). Furthermore, genistein upregulates the expression levels of SOD, CAT, glutathione, and GSH-Px in the cells. Zhang et al. [23] found that genistein-mediated oxidative damage protection was via SIRT1 stimulation, which deacetylated FOXO3a. Poljsak and Milisay [24] suggested that NAD^+^’s role as a signaling molecule influenced transcription factors, helped SIRTs convert ROS, and influenced direct apoptotic response to ROS production. Diet modulators stimulate multiple SIRT family genes and offer protection against oxidative stress.

### 2.3. SIRTs and Apoptosis

Apoptosis maintains cell balance in the human body. The process genetically detects abnormal cells and initiates their death [25,26]. The increased permeabilization of the mitochondrial outer membrane, caspase-activating molecules, caspase-independent death effectors, and the disruption of ATP production trigger programmed cell death. Once started, cell death becomes inevitable [27]. The apoptosis process involves cell turnover, new structure development, the immune system, hormone-dependent atrophy, embryonic development, chemical-induced death, and the progression of several diseases [27]. SIRT activation can regulate the apoptosis process. Quercetin treatment alleviates isoniazid-induced hepatotoxicity through the SIRT1/ERK signaling pathway in HepG2 cells. Quercetin reversed isoniazid-induced SIRT1 inhibition, upregulated Bcl-2 expression levels, downregulated Bax, cleaved caspase-3, and cleaved caspase-9 expression levels in [28].

Liang et al. [29] found that lipid accumulation was reduced due to combined genistein and daidzein stimulating colon cancer cell apoptosis. The perilipin-1, ADRP, and Tip-47 family protein expression levels were downregulated by a genistein–daidzein mixture. Meanwhile, expression levels of PPARγ, Fas, FABP, glycerol-3-phosphate acyltransferase, and microsomal TG transfer protein were significantly induced. The genistein–daidzein mixture induced cell apoptosis through significantly increasing FOXO3a and caspase-8 expression and decreasing P13k expression. The activation of the SIRT3 gene decreased stress-induced apoptosis via Bcl2-53 and JNK regulation. Studies showed that the SIRT3 gene suppressed tumor cells via ROS repression and DNA protection [30]. SIRT3-null mice could develop mammary tumors after one year; meanwhile, SIRT3 expression levels of breast cancers were decreased in humans.

### 2.4. SIRTs and Autophagy

Autophagy functions as a cellular housekeeper process: it degrades the bulk of defective organelles, aging cells, pathogens, and proteins in eukaryotes. The autophagy process directs the formation of a double-membrane cytoplasmic vesicle and autophagosomes come from the cell’s endoplasmic reticulum [31]. The formed vesicle engulfs target substances, fuses to the lysosome, and degrades them. The efficiency and maintenance of the autophagy process enhance lifespan. Energy-deficient conditions activate the AMPK pathway while stress conditions activate the ULK1 cascade; these regulate NAD^+^ levels and metabolism and promote autophagy [32]. CR induces SIRT1 activation and p53 deacetylation, which regulate autophagy. In studies by Yang et al. [33], resveratrol treatment could activate SIRT1 to prevent osteoporosis in aging rats. In vivo results showed that resveratrol-mediated SIRT1 and P13k/Akt/mTOR signaling pathways could significantly improve bone quality and protected osteoblasts in rats with osteoporosis.

SIRT1 induces autophagy via two modes. The first deacetylates light chain 3 (LC3), which binds to autophagy proteins (Atg 7 and Atg 8) [34]. The alternative model is REGy, which prevents SIRT1 from binding and deacetylating autophagy complex components [35]. SIRT2 binds to forkhead box O1 (FOXO1) and deacetylates it, interacting with Atg 7 [36,37]. SIRT2 KO mice manifest altered mitochondrial protein acetylation, reduced ATP production, defective mitophagy, increased oxidative stress, and increased p62, PINK1/Parkin, and ubiquitin-protein expression levels [37,38]. Mitochondrial functions are regulated by the SIRT3-activated AMPK/PGC-1α pathway. The SIRT3 promotes autophagy by upregulating mTOC1 expression and MnSOD production [39,40]. The overexpression of SIRT6 induces autophagy [41]. The autophagy process declines with age, resulting in certain disease conditions [31]. However, experts opine that targeting SIRTs could regulate these age-related degenerative diseases.

### 2.5. SIRTs and Aging

Aging is a functional decline of multi-cellular homeostasis pathways—including events such as genome fidelity, nutrient sensing, and proteostasis [42]. Aging ultimately culminates in the G1 phase arrest of the cell cycle process of continuously proliferating cells in response to either metabolic, genotoxic, or oncogene-induced stresses. Function decline puts cells at risk of malignant transformation via secreted degradative proteases, growth factors, and inflammatory cytokines. These secretions could compromise the microenvironments of non-senescent cells and promote cell cycle arrest, which are related with increases in senescence-associated β-galactosidase activity and DNA damage [13,43]. Pizarro et al. [44] evaluated the effect of resveratrol on neuroblastoma cell B65 in vitro. They found that resveratrol could inhibit cell proliferation and arrested cancer cells at the S phase. Resveratrol plays an anti-aging role by increasing expression of SIRT1. Similarly, calorie restriction conditions could also stimulate SIRT1 expression, whose levels coincided with NAD^+^ availability and reduced DNA damage in non-senescent cells, as discussed in [45]. Yousefzadeh and colleagues [46] studied the anti-aging effect of fisetin in aging mice. They found that fisetin could reduce senescence-related markers and improved tissue homeostasis; fisetin could also suppress age-related pathological alterations and extended the lifespan of mice.

The SIRT family proteins are a prominent and promising target and diagnostic tool in aging and anti-aging studies. Lee [36] stated that the upregulated expressions of SIRT1 could delay aging and increased lifespan. Elevated expression of SIRT2 is also a biomarker of senescence in cells [47]. SIRT3 gene deficiency could increase cellular ROS levels and damaged DNA molecules [48]. Additionally, the lack of the SIRT6 gene could promote the expression of glucose transporter type (GLUT) 1 and 4 transporters, which could cause hypoglycemia and premature death in mice [49]. Conversely, the overexpression of SIRT6 could increase IGF-1 binding proteins and change the phosphorylation levels of IGF-1 signaling components, which inhibits the IGF-1 pathway. These changes facilitate glucose tolerance, decrease fat accumulation, and extend lifespans of male mice [50]. Other investigators found that increased expression of SIRT7 mRNA is found in metabolically active tissues, while decreased expression is correlated with age [16]. Further, SIRT7-deficient cells display increased replication stress and impaired DNA repair [51].

### 2.6. SIRTs and Inflammation

Acute inflammation mends and restores cellular functions after a challenge, but chronic inflammation results in endothelial dysfunction, pro-inflammatory cytokine recruitment, adhesion molecules, matrix-degrading enzymes at the inflamed sites, and disease conditions [52]. NAD^+^ precursors have anti-inflammatory effects. SIRT transcription/protein and NAD^+^ levels are persistently reduced in tissues with chronic inflammation [53,54], making their increased activation an important anti-inflammatory biomarker. Quercetin alleviated diabetes-induced atherosclerosis-related inflammatory and oxidative stress in male Wistar rats. After two weeks of administering quercetin (30 mg/kg/day), they improved the lipid profile, vascular oxidative stress markers, and inflammatory cytokines in rats’ tissues. Quercetin ameliorated inflammatory and oxidative conditions through the AMPK/SIRT1/NF-kB pathway [55]. Additionally, quercetin relieved oxLDL, which caused endothelial oxidative injuries by stimulating the SIRT1 and AMPK/NF-kB/NADH oxidase/ATK/endothelial NO synthase signaling pathways [56].

Another study used piceatannol and resveratrol (10 mg/kg/day) in a high-fat diet-induced inflammation intervention experiment with male C57BL/6J mice. Results showed that SIRT modulators improved glucose and glycemic levels via increasing insulin receptor and AMPK levels in liver cells after four weeks [41]. Piceatannol administration increased the expression levels of SIRT1, SIRT3, SIRT6, PGC-1α, and forkhead box O1 genes. Resveratrol administration decreased the expression levels of IL-1β and IL-6 genes. SIRT modulators significantly decreased TNF-α expression levels. Nuclear-localized SIRTs (1, 2, 6, and 7) suppressed the activity and expression of inflammatory factor NF-κB by activating the cAMP/PKA/AMPK/SIRT1 signaling pathway [53,57]. Suppressed NF-κB activation could decrease the expressions of COX-2, iNOS, TNF-α, IL-1β, IL-6, and IL-8 but could increase the expressions of antioxidant and anti-apoptotic genes [54]. The activation of SIRTs suppresses pro-inflammatory cytokines and anti-apoptotic gene expression levels by deacetylating the NF-κB pathway p65 subunit [54].

### 2.7. SIRTs and Viral Infection

Viruses depend on host cells’ metabolism and compartments for energy, genetic components, replication, maturation, and dissemination. Reduced NAD^+^ cellular levels influence immune cell activation and infection advancement. Koyuncu et al. [58] demonstrated that small interfering RNA (siRNA)-mediated knockdown of individual SIRTs and drug-mediated inhibitory actions on SIRT enzymes increased viral progeny production in infected humans’ cells. They concluded that all SIRTs have a broad range of anti-viral effects. p53 and transcription factor c-Myc are associated with viral infections. SIRT1 activation of p53 leads to the apoptosis of infected cells. At the same time, deactivating SIRT2 degrades the c-Myc transcription factor and curbs viral nucleic acid biosynthesis. SIRT1 controls ACE2 receptor expression, which can be used for viral attachment. Dietary SIRT1 inhibitors deregulate ACE2 receptor proteins and inhibit viral attachment [19].

Pre-treating MERS-CoV-infected Vero E6 cells with resveratrol decreased nucleocapsid protein expression and downregulated apoptosis in a dose-dependent manner [59]. The N protein is essential for MERS-CoV replication. Recently, Saeedi-Boroujeni and Mahmoudian-Sani [60] reported that quercetin administration ameliorated COVID-19-positive-associated inflammatory conditions. Quercetin could suppress the NLRP3 inflammasome through SIRT1 activation and generated active forms of pro-inflammatory cytokines IL-1β and IL-18. SIRT1, SIRT2, and SIRT6 could inhibit viral infection-associated pro-inflammatory cytokine storm through deacetylating the NF-*κ*B p65 subunit. Natural modulators of SIRTs are similar to broad-spectrum antibiotics: (1) they can effectively prevent attachment, entry, and multiplication of many DNA and RNA viruses, (2) they are non-discriminatory against mutated variants because they predominantly interact with host cells and receptors, and (3) they suppress the expression of infection-associated pro-inflammatory cytokines [19,61].

### 2.8. SIRTs and Energy Homeostasis

The body auto-maintains its serum glucose balance through gluconeogenesis. SIRTs regulate hepatic gluconeogenesis and fatty acid oxidative pathways by targeting and deacetylating the PGC-1α protein [62]. A study by Higashida et al. [63] on muscular mitochondrial biogenesis showed that a high concentration of resveratrol lowered ATP concentration and activated the AMPK/SIRT1 pathway in the skeletal muscle of mice. Resveratrol improved the mice’s running endurance through SIRT1-mediated deacetylation, activating PGC-1α and increasing mitochondrial proteins. Resveratrol administration could increase SIRT1 expression and suppressed PGC-1α expression. It could also lower mitochondrial biogenesis and attenuated tesaglitazar-induced cardiac dysfunction in mice models [64]. Overexpression of the SIRT2 gene could inhibit preadipocyte differentiation, while decreased expression promotes adipogenesis in 3T3-L1 cells [65].

A lack of SIRT3 results in fatty acid disorders and lowers ATP levels during fasting of mice because SIRT3 influences ATP formation and adaptive thermogenesis [66]. SIRTs regulate the expression and maturation of adipocytes. They also modulate plasma glucose levels, mitochondrial energy capacity, insulin secretion, insulin tissue sensitivity, and cholesterol/lipid homeostasis [67,68]. The activations of PPARs and PGC-1α modulated by SIRT1 significantly affect fat mobilization and fatty acid oxidation [69]. The combined effect of berberine and resveratrol resulted in one-fold higher lipid metabolism via significantly increased low-density lipoprotein receptor expression in HepG2 cells [70]. Resveratrol showed better synergistic results with other Sirtfood components.

### 2.9. SIRTs and Cancer

One-third of all cancer-induced deaths can be averted by lifestyle change—including the consumption of proper nutrition, according to Danaei et al. [71]. However, multi-risk factors increase cancer cases and plague cancer studies. Cancerous cells propagate out of control and cause dysfunctions in nearby normal cells. SIRTs are the primary metabolic and stress sensors. SIRT2 and SIRT6 suppress oncogenic factors, while the SIRT1 gene has bifunctional actions [72,73]. A retrospective study by Chao et al. [74] surmised that resveratrol activated SIRT1 in human chondrosarcoma cells, decreased cell viability, and induced apoptosis dose-dependently. The mechanism of resveratrol exerting its underlying inhibitory effect was the deacetylation of the p65 subunit of the NF-kB factor. Resveratrol activated SIRT-mediated Akt, PI3K/Akt, NF-κB, and ER signaling pathways to elicit its anti-cancer function [75].

Genistein could suppress tyrosine kinases and regulated Atk/MEK signaling pathways, which caused cell cycle arrest and inhibited the proliferation of cancer cells [76]. Additionally, SIRT modulators ameliorate cellular stress through their high antioxidative effects. They activate the Nrf2 pathway to scavenge radicals and produce antioxidative enzymes [77]. Biological evidence implicates mitochondrial SIRTs (mtSIRTs) as essential regulators that control cancer and tumor cell progression or ‘onco-metabolism’. SIRTs 3, 4, and 5 target and alter metabolic and mitochondrial energetics of cancer cells. These genes elicit pathways that control cell proliferation, apoptosis, cell cycle progression, inflammation, angiogenesis, invasion, and metastasis [72]. The multi-functions of SIRT modulators make them suitable natural therapeutics.

## 3. Sirtfoods and SIRT Modulations

### 3.1. The Relationship between Sirtfoods and Gene Modulation

The links between natural SIRT modulators and their bioactivities lie in the bioavailability of NAD^+^. Studies showed that SIRTs consumed depended on the rate-limiting NAD^+^ to simulate CR-related pathways in aging mice models, diabetic rodents, and human experiments [8,78]. The NADH molecule is the predominant electron donor in the ETC, and the NADH/NAD^+^ redox potential indicates the bioenergetic status in a functional cell [78]. NAD^+^ also co-substrates for SIRTmediated lysine acetylation, succinylation, ADP-ribosylation, and malonylation. NAD is cleaved to ADP-ribose and NAM. The latter inhibits SIRT bioactivities—similar to a feedback reaction [79,80].

SIRT foods simulate the CR condition and reduce nutrient intake by 20–40% without causing malnutrition [10,81]. The CR-stimulated poly-pharmacological activities regulate epigenetic mechanisms, target numerous substrates, and initiate a two-way interactive reaction between dietary and gut environmental components [66,82]. The earliest mention of ”Sirtfoods” was by Pallauf et al. [7]. They proposed combing Mediterranean and Asian diets. The ”MediterrAsian” diet pattern had a small portion of non-plant components and a significant portion of plant-based low-dense energy and health-promoting SIRT modulators. However, progenitors of modern Sirtfoods™ claim that their plant-only recipe “turns on consumers’ skinny genes”, which regulate metabolic disorders and improve lifespans [6].

Skeptics have valid concerns. The costs of Sirtfoods™ recipe books and juice extractors are high, cutting off potential consumers. The ”narrow” nutrient profile or low-calorie diet (1000–1500 cal/day) and absence of other essential nutrients may cause hunger or malnourishment [6]. The narrow nutrient profile is a serious problem because consumers must follow a three-week-long consumption regime. Therefore, in-depth evaluations will settle acceptability concerns, set universally acceptable doses, and elucidate synergistic impacts of Sirtfoods™.

### 3.2. Natural Compounds Modulate SIRTs

The most studied SIRT modulators are polyphenols, which are pharmacologically more effective and safer than synthetic activators of SIRTs [21]. Sirtfoods™ comprise proprietary recipes for twenty kinds of fruits and vegetables: arugula (rocket), bird’s eye chili, blueberries, buckwheat, capers, cereals, coffee, dark chocolate (85% cocoa), extra-virgin olive oil, kale, lovage, matcha green tea, Medjool dates, onions, parsley, red chicory, red wine, soy, strawberries, turmeric, and walnuts [6]. Extensive mechanistic and structural studies conclusively establish that Sirtfoods’ modulating compounds interact directly with SIRT1 and other SIRT isoforms [83]; see Table 1.

Data suggest that SIRT-activating compounds bind to the SIRTs, resulting in a composite site that enhances substrate binding and yields an activating effect [83]. Conversely, the actions of SIRT inhibitors are equally important, especially in cancer, neurodegenerative diseases, and viral infection treatments: they target the polypeptide-binding cleft and the NAD^+^ pocket for competitive inhibition [19,83,84]. The synergistic potentials of SIRT family genes are promising and may inform the emergence of the modern Sirtfoods pattern [6,84]. Connell et al. [78] submitted a valid question—”are biomolecular SIRT modulators the ’silver bullets‘ of natural therapeutics and functional foods?“ The answer lies in more pre-clinical trials. Experts must determine the sustainable dosage, mix, and synergistic modulating effects per therapy.

## 4. Mechanisms of Sirtfoods

### 4.1. The Gut Environment

The gut environment is a vital ”organ” in transforming SIRT modulators. Significant differences exist between the functions and compositions of a healthy (eubiosis, stable, lean) and an unhealthy (dysbiosis, unstable, obese) gut pattern. Table 2 lists the different microorganisms that can transform the active components of Sirtfoods into metabolites with biological functions. Authors Graf et al. [85] and Hold [86] demonstrated the importance of diet–gut microbiome interactions in clinical trials amongst African and American adolescents, respectively. Their submissions attested that the modern Sirtfoods^TM^ shed the three-week efficacy claims because of ”long-term consumption benefits” on the gut structure. Short-term benefits are easily reversible—only sustained stimulation of the gut makes for a “new normal” [87,88].

### 4.2. Abridged Sirtfoods’ Metabolisms

**Figure 2:**
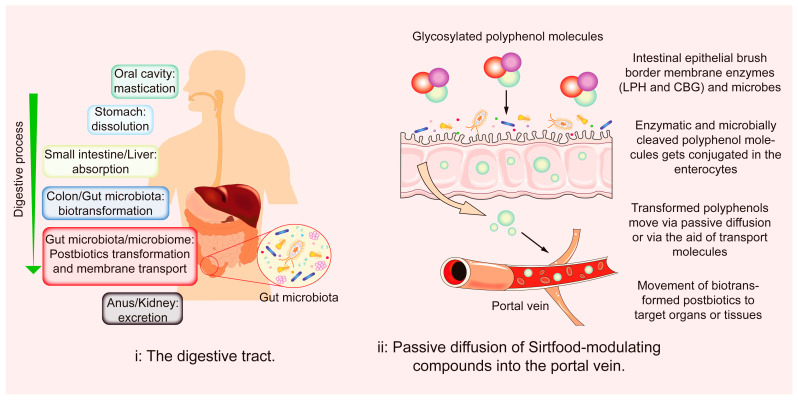
Process overview for Sirtfood components’ modulation of SIRTs genes.

**Figure 3:**
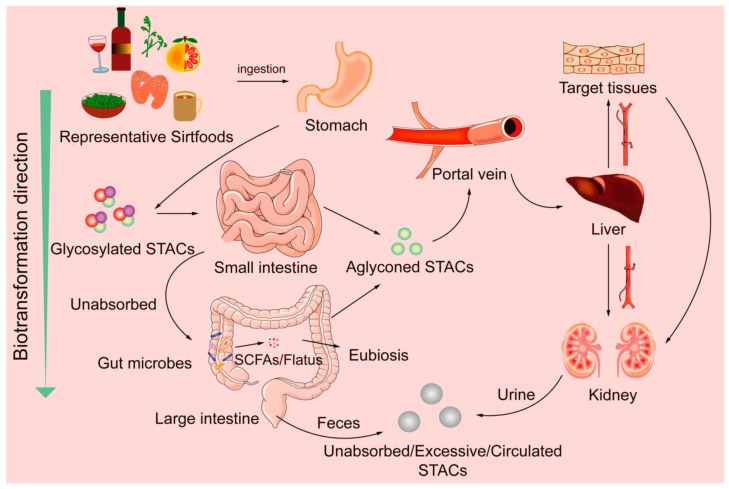
Overview of STACs’ biotransformation processes.

Sirtfoods, being plant-based, have antioxidative properties coupled with the reactivity of their gut-induced metabolites. A significant proportion of polyphenols released from the food matrix is excreted and does not reach the target organs. The low bioavailability of polyphenols is influenced by interrelated factors. These factors include the number of polyphenols released from the food matrix, food processes, genetics, and gut microbes [90]. Only a tiny (5–10%) proportion of polyphenols released from the food matrix are absorbed in the small intestine [102]. Polyphenols are transformed in enterocytes before entering the bloodstream. However, low-molecular-weight polyphenols and anthocyanidins are readily absorbed in the stomach [103,104]. Conversely, 95–90% reach the large intestine unaltered, but they can modulate microbiota diversity and change metabolic productions [102,103,105]. Figure 2 and Figure 3 are overviews of polyphenol biotransformation processes. Phase II metabolites are further deconjugated and metabolized by glucuronidases and sulfatases in the liver. They circulate to targeted organs/tissues via the bloodstream [103,104,106]. The gut–diet interaction increases postbiotic concentration and activeness higher than their parent compound [102,106]. Table 2 lists natural SIRT modulators, postbiotics, health benefits, and associated microbes.

The starch/fiber portions of Sirtfoods are also quantifiable, absorbable, and gut transformable. After ingestion, available starch is readily digested in the small intestine, while colonic microbiota ferment resistant starch portions and food cell wall polysaccharides to yield varying end-products [91,107]. Fermentation end-products such as SCFAs (acetic, propionic, and butyric acids) and flatus (carbon dioxide, hydrogen, methane, nitrogen, oxygen, hydrogen sulfide, indole, skatole, and volatile amines) [91,108] are a mix of energy fuels and health-promoting agents. Table 2 presents Sirtfood sources and components, their postbiotics, and the microorganisms involved in their metabolisms [9,85,88,89,90,91,92,93,94,95,96,97,98,99,100].

Glycosylated polyphenols are cleaved in the intestine by gut microbiota and epithelial enzymes (CBG and LPH). Conjugated metabolites pass through the epithelial cells, via passive diffusion, into the portal vein and then to the liver, where they undergo further metabolism. Enterohepatic recirculation of conjugated aglycons occur, after which they are deconjugated by gut microbes and reabsorbed into the bloodstream via enterocytes.

### 4.3. Bioactivity Regulations of SIRTs

The components of Sirtfoods are often detected as phase II metabolites and go on to modulate SIRTs. For example, SIRT1 represses transcription by docking to receptors such as the nuclear receptor corepressor (NCoR) and silencing the mediator for the retinoid and thyroid hormone receptor (SMRT). The SIRT1/NCoR/SMRT complex binds to the PPARδ response elements and represses PPARδ target gene expressions [101]. Figure 1 shows interlinked pathways elicited by Sirtfoods components via SIRT1 activation. Although SIRT1 is the most studied, other SIRT isoforms have similar functions: (1) Physiological conditions: low energy levels stimulate SIRTs. At the same time, high energy levels repress their activities. (2) The bioavailability of SIRT co-factor NAD^+^ also stimulates SIRT expressions: An increase in NAD^+^ level increases SIRT bioactivities. (3) Complex formation with other proteins: SIRTs are regulated positively by the active regulator of SIRT1 (AROS) and negatively by NCoR1 and SMRT [82].

## 5. Conclusions and Perspectives

The preponderance of data shows that Sirtfoods elicit multi-pharmacological and pleiotropic health functions that mitigate metabolic disorders. Therefore, whether as a single extract or combined, Sirtfoods can bridge ”what we consume” and ”health.“ Plant-based meals are natural, low-cost, and have long-term health benefits. Wholesome Sirtfoods target and regulate genes to promote health, thus attracting increased interest in applying them as functional foods. Therefore, future in vivo and clinical trials should elucidate potential issues and benefits associated with Sirtfoods and determine their pharmacokinetics, pharmacodynamics, synergistic gut interactions, benefits, and appropriate dose. Objective evaluation is essential because different modulators either activate or inhibit SIRT activities. Proven data will avert unforeseen adverse physiological outcomes for consumers—the keyword is balanced diets.

Food authorities should sanction dietary patterns or recipes with unsubstantiated health claims. Formulated dietary patterns should be verified anew, as not all experimental results are clinically translatable. Scientists should tackle diet issues such as low bioavailability and rapid metabolism of dietary polyphenols.

## Figures and Tables

**Table 1:** SIRTs family genes: localization, substrates, functions, and modulators.

Type (Class)	Localization (Mol. Weight)	Tissue Expression	Enzyme Activity	Substrates	Functions	Activators	Inhibitors
SIRT1 (I)	Nuclear; cytosolic. (81.7 kDa)	Brain; Skeletal muscle; Heart; Kidney; Uterus.	NAD^+^-dependent deacetylases catalyze the deacetylation of histones and nonhistone proteins.	Acetyl-CoA synthetase 1;Akt; Androgene receptor; APE1; Atg 5; Atg 7; Atg 8; BCL6; BMAL1; c-MYC; cortactin; CRABPII; CRTC2; DNMT1; eNOS; FOXA2; FOXO family; FXR; HIF1α; HIF2α; Histone H1K26; Histone H3K9; Histone H4K16; Histone K14; Histone K56; HIV Tat; HMGB1; HMGCS1; hMOF; HSF1; IRF-1; KAP1; KDAC1; Ku70; LKB1; LXR; MEF2; NBS1; NF-κB(p65); Notch1; p300; p53; p73; PARP1; PCAF; PER2; PGAM1; PGC1α; PML; PPAR-γ; RFX-5; SATB1; Smad7; SREBP-1c; SUV39H1; TDG; TFAM; TIP60; TopBP1; TORC1; UCP2; WRN; XPA.	Apoptosis; Cell cycle; Cell migration; Cell survival; Circadian control; Inflammation; Insulin signaling; Lifespan regulation; Metabolism regulation; Neuroprotection; Oxidative stress response; Mitohormesis; Tumor suppression; Viral transcription.	Resveratrol; Piceatannol; Quercetin; Fisetin; Daidzein; Genistein; Berberine; Flavonoid mulberrin; The xanthone gartanin; The alkaloids quinidine and quinine.	Tanikolide dimer; Chalcones; Biochalcones.
SIRT2 (I)	Nuclear; cytosolic. (41.5 kDa)	Brain.	NAD^+^-dependent HDAC and ADP-ribosyl transferase activities.	Akt; ALDH1A1; APCCDH1; ATRIP; BubR1; CDC20; CDK9; FOXO1; FOXO3a; G6PD; H4K16; HIF1α; Histone H3K18; Histone H3K56; Histone H4K16; K-RAS; NF-κB (p65); p300; p53; Par-3; PEPCK1; PGAM; TUG; α-tubulin; β-secretase 1.	Adipocyte differentiation; Autophagy; Bacterial infections; Cell cycle regulation; Energy homeostasis Fibrosis; Genome stability; Immune response; Longevity; Myelination; Neurodegeneration.	Resveratrol.	Tanikolide dimer.
SIRT3 (I)	Mitochondrial; Nuclear. (43.6 kDa)	Brain; Heart; Liver; Kidney; Brown adipose tissue.	NAD^+^-dependent deacetylases catalyze the deacetylation of histones and nonhistone proteins.	Acetyl-CoA synthetase 2; Aconitase 2; ALDH2; ATP-synthase F1; Complex 1; Cyclophilin D; FOXO3a; GDH; GOT2; GSK3β; Histone H4K16; HMGCS2; Hsp10; Long-chain acyl-CoA dehydrogenase; HMGCS; Isocitrate dehydrogenase 2; Ku70; LCAD; LKB1; MDH; Mitochondrial ribosomal protein L10; MnSOD; OGG1; OPA1; OTC; PDH; PGC-1α; SDH; Skp2; SOD2; UCP-1; VLCAD.	Fatty acid oxidation; Fibrosis; Longevity; Metabolism regulation; Oxidative phosphorylation; Oxidative stress TCA cycle; Thermogenesis; Urea cycle.	Resveratrol; Trans-(−)-ε-viniferin; Piceatannol.	-
SIRT4 (II)	Mitochondrial (35.2 kDa)	Brain; Liver; Kidney; Heart; Pancreatic β-cells.	NAD^+^-dependent HDAC and ADP-ribosyl transferase activities.	ANT2/3; GDH; Hsp60; MCD; Pyruvate dehydrogenase complex; Stress-70.	Fatty acid oxidation; Insulin secretion; Metabolism regulation; TCA cycle; Tumor suppression.		-
SIRT5 (III)	Mitochondrial; Nuclear (33.9 kDa)	Brain; Heart; Muscle; Testis; Lymphoblast.	NAD^+^-dependent deacetylases catalyze the deacetylation of histones; Desuccinylase; Demalonylase; Deglutarylase.	Cytochrome c; Carbamoyl phosphate; GAPDH; HMGCS2; Hsp70; IDH; PML; Prx-1; SOD1; Synthetase 1; UOX; VLCAD.	Apoptosis; Fatty acid oxidation; Ketone body synthesis; Oxidative stress; Urea cycle.	Resveratrol; Piceatannol.	-
SIRT6 (IV)	Nuclear (39.1 kDa)	Brain; Heart; Muscles; Ovaries; Bone cells.	NAD^+^-dependent ADP ribosyltransferase (ART) mediating mitochondrial protein ribosylation; Demyristoylase; Depalmitoylase.	GCN5; Histone H3K9; Histone H3K56; H3k18ac; TNF-α; KAP1; NF-*κ*B; p70; PAPR1.	Apoptosis; DNA repair; Genome stability; Longevity; Protein secretion.	Anthocyanidins; Gallic acid derivatives.	Catechins; Epicatechins; Phytoestrogens.
SIRT7 (IV)	Nucleolus; Cytoplasm (44.9 kDa)	Peripheral blood cells; CD33^+^ myeloid bone marrow precursor cells.	NAD^+^ dependent deacetylase; Desuccinylase; Regulates the RNA polymerase I (Pol I) transcriptional machinery.	DNA-PK; GABPβ1; Histone H3K122; Histone H3K18; H3K36ac; p53; PAF53; RNA polymerase I; U3–55k.	Cell cycle regulation; Genome stability; Regulation of rDNA transcription; Tumor promotion.		-

**Table 2:** Sirtfood components, postbiotics, and microbes involved.

	Polyphenol	Diet Source	Postbiotics	Health Proffering Mechanisms	Microbes Involved	References
1	Resveratrol (non-flavonoid stilbene)	Red wine, peanuts, red grapes, and selected teas.	Dihydroresveratrol, Lunularin, 3,4′-dihydroxy-trans-stilbene.	AMPK activity ↑→lipid lowering TG plasma ↓ Carcinogenesis ↓ Bacteroidetes↑ SCFAs ↑	*Bacteroidetes, Actinobacteria, Verrucomicrobia, Cyanobacteria, Slackia equolifaciens,* and *Adlercreutzia equolifaciens.*	[88].
2	Trans-(−)-ε-viniferin (Stilbene)	Grapes and wines.	ε-viniferin mono-sulfate, ε-viniferin mono-glucuronide.	Anti-fungal ↑ Intestinal sodium-dependent glucose uptake via the SGLT1↓	*Enterobacteriales.*	[89].
3	Quercetin (dietary flavonols)	Tea, red wine, berries, apples, tomatoes, beans, and onions.	Quercetin-3-glucuronide, Quercetin-7-glucuronide, 3,4-dihydroxyphenylacetic acid, 3-(3-hydroxyphenyl) propionic acid, 3,4-dihydroxybenzoic acid, 4-hydroxybenzoic acid.	Cellular barrier ↓ antioxidative pathway ↑. serum IL-6↓ Synthesis of polyamines ↓ Anti-inflammation ↑ Bacteroidetes ↑ SCFAs ↑.	*Eubacterium ramulus, Eubacterium oxidoreducens, Clostridium orbiscindens,* and *Butyrovibrio* spp.	[90].
4	Fisetin (dietary flavonol)	Strawberries, apples, persimmons, grapes, peach, lotus root, cucumber, teas, onions, kiwi, and kale.	Glucuronidated fisetin, Geraldol (3,4′,7-trihydroxy-3′-methoxyflavone), and Glucuronidated geraldol.	Anti-pathogenic ↑ Butyrate production ↑ Apoptosis ↑ MAPK/NF-*κ*B ↓.	*Lachnospiraceae.*	[91].
5	Piceatannol (Stilbene)	Grapes, passionfruit, white tea, wines, Japanese knotweed, Asian legumes, and Korean rhubarb.	Piceatannol disulfate, piceatannol monosulfate-1, and piceatannol monosulfate-2.	Cell survival or proliferation ↓ Cell cytotoxicity ↑ Reactive oxygen species level ↓ Autophagy ↑ Cell cycle proteins ↑.	*Lactobacillus* spp, *Lachnospiraceae*, and *Bacillus megaterium* CYP102A1.	[89].
6	Daidzein (isoflavones)	Soybeans, legumes, whole grains, berries, and nuts.	Dihydrodaidzein; can further be converted to *S*-equol.	Hormone-dependent diseases ↓ Antioxidation ↑ Anti-cancer ↑.	*Bifidobacteria* sp. (*B. breve* and *B. longum), Lactococcus* strain 20–92, *Eggerthella* sp. Julong 732, and *Eubacterium limosum.*	[92].
7	Anthocyanidin	Berries, currants, grapes, colored leafy vegetables, grains, roots, and tubers.	4-hydroxybenzoic acid, protocatechuic acid, gallic acid, vanillic acid, syringic acid, catechol, pyrogallol, resorcinol, tyrosol, 3-(3′-hydroxyphenyl) propionic acid, dihydrocaffeic acid, and 3-(4′-hydroxyphenyl) lactic acid.	Modulation of gut microbiota → Anti-pathogenicity ↑ SCFAs ↑ Vitamin production ↑ Anti-inflammatory ↑ Gut microbiota metabolites ↑→ macrophage reverse cholesterol transport ↑ Atherosclerotic lesion ↓.	*Bifidobaterium* spp., *Lactobacillus* spp., *Eubacterium ramulus*, and *Clostridium saccbarogumia.*	[85,93].
8	Gallic acid derivatives	Grapes, gallnuts, pomegranates, and tea leaves.	Pyrogallol-1-*O*-glucuronide, 4-OMeGA, 4-OMeGA-3-*O*-sulfate, pyrogallol-*O*-sulfate, deoxypyrogallol-*O*-sulfate, and *O*-methylpyrogallol-*O*-sulfate.	Gut microbial modulation, *Helicobacter pylori* ↓ Anti-pathogenic ↑ Anti-cancer ↑ anti-inflammatory ↑ SCFAs ↑.	*Pseudomonas* and *Atopobium* spp.	[94].
9	Genistein (isoflavones)	Soy, raisins, currants, prunes, mango, passionfruit, quinoa seeds, and peanuts.	p-ethyl phenol and 4-hydroxy-phenyl-2-propionic acid.	Binding to estrogenic receptors → estrogenic/anti-estrogenic activities. Carcinogenesis ↓.	*Lactobacillum, Lactoccocus, Enterococcus, Bifidobacterium,* and *Bacteroides.*	[90].
10	Xanthone gartanin	Mangosteen.	Alpha- and *γ*-mangostin.	Apoptosis ↑ Akt, MAPK, NF-*κ*B pathways ↓ Anti-microbial ↑.	Enterobacteriaceae and Enterococcaceae.	[9].
11	Quinine	Cinchona tree bark.	*3-* *hydroxyquinine.*	Anti-malarial ↑ T2R family receptors ↑.	*Subdoligranulum* spp., *Akkermansia muciniphila, Roseburia inulinivorans*, *Methanobrevibacter smithii*, and *Roseburia intestinalis.*	[95].
12	Catechins	*Acacia catechu* L., *Camellia sinensis,* red wine, and chocolate.	UDP-glucuronosyltransferases (UGTs); sulphotransferases (SULTs); and catechol-O-methyltransferase (COMT).	Anti-cancer ↑ Antioxidation ↑ Anti-inflammatory activities ↑ Immune activity ↑ Receptor tyrosine kinase ↓.	*Aspergillus, Penicillium, Rhizopus, Mucor,* Yeasts, and Bacterium.	[96].
13	Epicatechins	Apples, blackberries, broad beans, cherries, grapes, pears, raspberries, dark chocolates, cocoa, and tea leaves.	1-(3′,4′-dihydroxyphenyl)-3-(2″,4″,6″-dihydroxyphenyl)-2-propanol (3,4-diHPP-2-ol) and 5-(3′,4′-dihydroxyphenyl)-γ-valerolactone (3,4-diHPV)	Antioxidant activities ↑ Modulates NO and ROS→improves arterial vessels endothelial functions.	*Eubacterium* SDG-2, *Lactobacillus plantarum, Eggerthella lenta*, and *Adlercreutzia equolifaciens*	[97].
14	Phytoestrogens	Flax seeds, grapes, soybeans, kidney beans, apples, cabbage, spinach, hops, garlic, onion, wine, and tea.	Equol, urolithins, and enterolignans.	Cardiovascular diseases ↓ Diabetes ↓ Breast cancer ↓ Osteoporosis ↓ Anti-inflammation ↑.	*Bacteroides*, *Clostridium* strains, *Lactobacillus* strains, *Gordonibacter urolithinfaciens, Gordonibacter pamelaeae* DSM 19378(T), *Bifidobacterium, Lactobacillus, Enterococcus faecalis*, and *Streptococcus bovis.*	[98].
15	Tanikolide dimer	*Lyngbya majuscule:* a Madagascar marine Cyanobacterium.	-	Anti-fungal activity against *Candida albicans* Anti-bacterial activity against *Mycobacterium smegmatis* and *Streptococcus pyogenes* Anti-cancer ↑.	*-*	[9].
16	Chalcones	Citruses, *Ochna* sp., apples, tomatoes, shallots, bean sprouts, and potatoes.	Dihydrochalcone	Anti-cancer ↑ Antioxidative ↑ Anti-microbial ↑ anti-inflammatory ↑.	* Eubacterium ramulus. *	[99].
17	Biochalcones	*Ochna* sp. and *Rhus pyroides.*	-	Anti-protozoal ↑ Anti-viral ↑.	* - *	[97].
18	Berberine	Chinese herb (*Rhizoma coptidis*).	Raisanberine and CPU86017.	Anti-viral ↑ Anti-cancer ↑ Antioxidative ↑; Anti-inflammation↑ Cardioprotective effects ↑.	*Clostridium hiranonis, C. scindens,* and *C. hylemonae.*	[99].
19	Undigested polysaccharides/ dietary fiber	SCFAs.	Acetate, propionate, and butyrate.	Colonocytes and epithelia cells Apoptosis ↑ Histone deacetylases ↑ →FFAR2 and FFAR3 →intestinal gluconeogenesis ↑ → satiety ↑ Glucose → adiposity ↓ Lipogenesis ↑ Gut microbial eubiosis↑.	*Bifidobacterium longum, Eubacterium hallii,* Lachnospiraceae, *Faecalibacterium prausnitzii,* Negativicutes, *Clostridium* species, *Roseburia inulinivorans, and Ruminococcus obeum.*	[100].
20	Undigested polysaccharides/dietary fiber	Gases.	Mercaptans, sulphated mucins, and hydrogen sulfide.	Microbial redox reactions ↑ Anaerobic fermentation ↑ Lubricate gastrointestinal tracts ↑ Cell signaling molecules ↑.	*Bacteroides, Clostridium, and Desulfovibrio.*	[101].

## Data Availability

No new data were created or analyzed in this study. Data sharing is not applicable to this article.

## Abbreviations

ACE2—angiotensin-converting enzyme 2; Ad5—adenovirus type 5; AMP—Adenosine monophosphate; AMPK—AMP-activated protein kinase; AROS—active regulator of SIRT1; Atg—autophagy proteins; ATP—adenine triphosphate; CAT—catalase; CD—catalytic domain; CR—calorie restriction; ER—endoplasmic reticulum; ERK—extracellular-regulated protein kinase; ETC—electron transport chain; FOXO—forkhead box O1; GLUT—glucose transporter type; GSH-Px—Glutathione peroxidase; HCMV—human cytomegalovirus; HSV1—herpes simplex virus 1; IGF-1—insulin-like growth factor 1; IL-6—interleukin-6; iNOS—inducible nitric oxide synthases; KO—knock out; MAR 1—mating-type regulator 1; MDA—malondialdehyde; MEK—mitogen-activated protein kinase/ERK kinase; MERS-CoV—the Middle East respiratory syndrome-related coronavirus; MnSOD—manganese superoxide dismutase; mTOR—mammalian target of rapamycin; mtSIRTs—mitochondrial SIRTs; NAD+—nicotinamide adenine dinucleotide; NADH—nicotinamide adenine dinucleotide hydrogen; NAM—nicotinamide; NCoR1—nuclear receptor corepressor1; NLRP3—NLR family pyrin domain containing 3; NO—nitric oxide; OAADPr—2-3-O-acetyl-ADP-ribose; PGC-1α—peroxisome proliferator-activated receptor gamma coactivator 1-alpha; PPAR—peroxisome proliferator-activated receptor; ROS—reactive oxygen species; sir 2—silent information regulator 2; siRNA—small interfering RNA; SIRT—sirtuin; SMRT—silencing mediator of retinoid and thyroid hormone receptors; SOD—superoxide dismutases; TNF-α—Tumor necrosis factor-alpha.
